# The genome sequence of the jet ant, *Lasius fuliginosus *(Latreille, 1798)

**DOI:** 10.12688/wellcomeopenres.23347.1

**Published:** 2024-11-08

**Authors:** Liam M. Crowley, University of Oxford and Wytham Woods Genome Acquisition Lab, Darwin Tree of Life Barcoding collective, Wellcome Sanger Institute Tree of Life Management, Samples and Laboratory team, Wellcome Sanger Institute Scientific Operations: Sequencing Operations, Wellcome Sanger Institute Tree of Life Core Informatics team, Tree of Life Core Informatics collective, Darwin Tree of Life Consortium

**Affiliations:** 1 University of Oxford, Oxford, England, UK

**Keywords:** Lasius fuliginosus, jet ant, genome sequence, chromosomal, Hymenoptera

## Abstract

We present a genome assembly from an individual female *Lasius fuliginosus* (the jet ant; Arthropoda; Insecta; Hymenoptera; Formicidae). The genome sequence is 256.2 megabases in span. Most of the assembly is scaffolded into 14 chromosomal pseudomolecules. The mitochondrial genome has also been assembled and is 18.75 kilobases in length.

## Species taxonomy

Eukaryota; Opisthokonta; Metazoa; Eumetazoa; Bilateria; Protostomia; Ecdysozoa; Panarthropoda; Arthropoda; Mandibulata; Pancrustacea; Hexapoda; Insecta; Dicondylia; Pterygota; Neoptera; Endopterygota; Hymenoptera; Apocrita; Aculeata; Formicoidea; Formicidae; Formicinae; Lasiini; *Lasius; Dendrolasius*; *Lasius fuliginosus* (Latreille, 1798) (NCBI:txid231986).

## Background

*Lasius fuliginosus*, commonly known as the jet ant or jet black ant, is a species within the family Formicidae and subfamily Formicinae. It is characterized by a shiny black exoskeleton and medium-sized workers measuring approximately 4–6 mm in length. The glossy appearance and smooth body surface distinguish it from other species within the genus *Lasius* ( AntWiki, 2024).

This species is distributed across Europe and parts of Asia ( GBIF Secretariat, 2023). In the United Kingdom, *L. fuliginosus* is relatively widespread, particularly in southern and central regions ( Hoy, 1997). It predominantly inhabits deciduous woodlands and areas rich in decaying wood, which provides ideal conditions for nesting. The ants construct their nests in tree trunks, stumps, or fallen logs, creating intricate carton-like structures made from chewed wood fibres bonded with saliva.

During the early stages of colony formation, *L. fuliginosus* exhibits temporary social parasitism. A young queen infiltrates the nest of a closely related *Lasius* species, such as *Lasius umbratus*, *Lasius mixtus*, or others, by replacing the resident queen ( AntWiki, 2024). The host workers, unaware of the change, continue to care for the brood. Eventually, the colony becomes entirely composed of *L. fuliginosus* individuals. This strategy allows the queen to bypass the vulnerable phase of establishing a new colony independently.

Colonies of *L. fuliginosus* are highly organised and can consist of thousands of individuals led by a single queen. Workers are responsible for foraging, nest maintenance, and brood care. The species primarily feeds on honeydew produced by aphids and other sap-sucking insects, which they tend and protect in a mutualistic relationship ( AntWiki, 2024).

In this data note, we present a chromosomally complete genome sequence of *Lasius fuliginosus*, based on a female specimen collected from Dry Sandford Pit, Oxfordshire, UK. This sequencing effort is part of the Darwin Tree of Life Project, a collaborative initiative aiming to sequence all named eukaryotic species in the Atlantic Archipelago of Britain and Ireland ( Darwin Tree of Life Project Consortium, 2022).

## Genome sequence report

**Figure 1.**
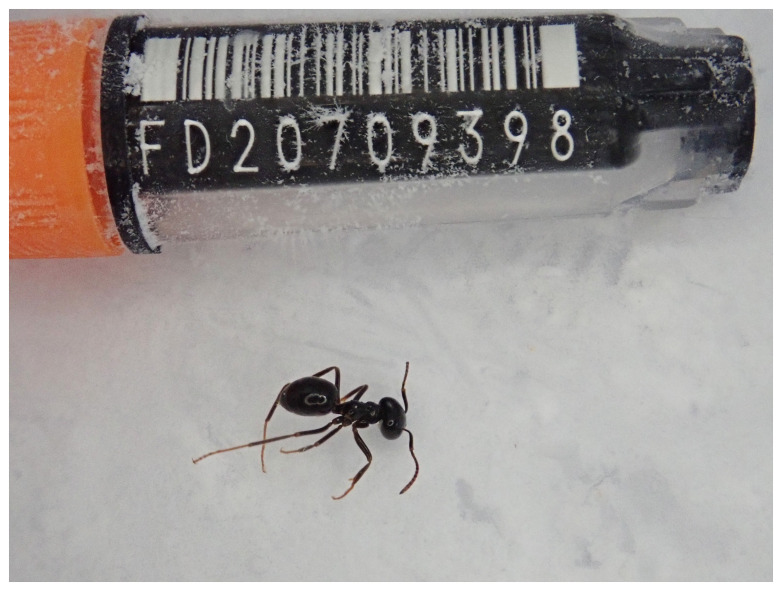
Photograph of the *Lasius fuliginosus* (iyLasFuli2) specimen used for genome sequencing.

The genome was sequenced from an adult female *Lasius fuliginosus* ( Figure 1) collected from Dry Sandford Pit, Oxfordshire, UK (51.69, –1.32) using Pacific Biosciences single-molecule HiFi long reads, generating a total of 22.02 Gb (gigabases) from 2.47 million reads, providing an estimated 121-fold coverage. Primary assembly contigs were scaffolded with chromosome conformation Hi-C data, which produced 178.07 Gb from 1,179.24 million reads. Manual assembly curation corrected 212 missing joins or mis-joins and removed 13 haplotypic duplications, reducing the assembly length by 0.42% and the scaffold number by 19.84%, and decreasing the scaffold N50 by 1.63%.

**Figure 2.**
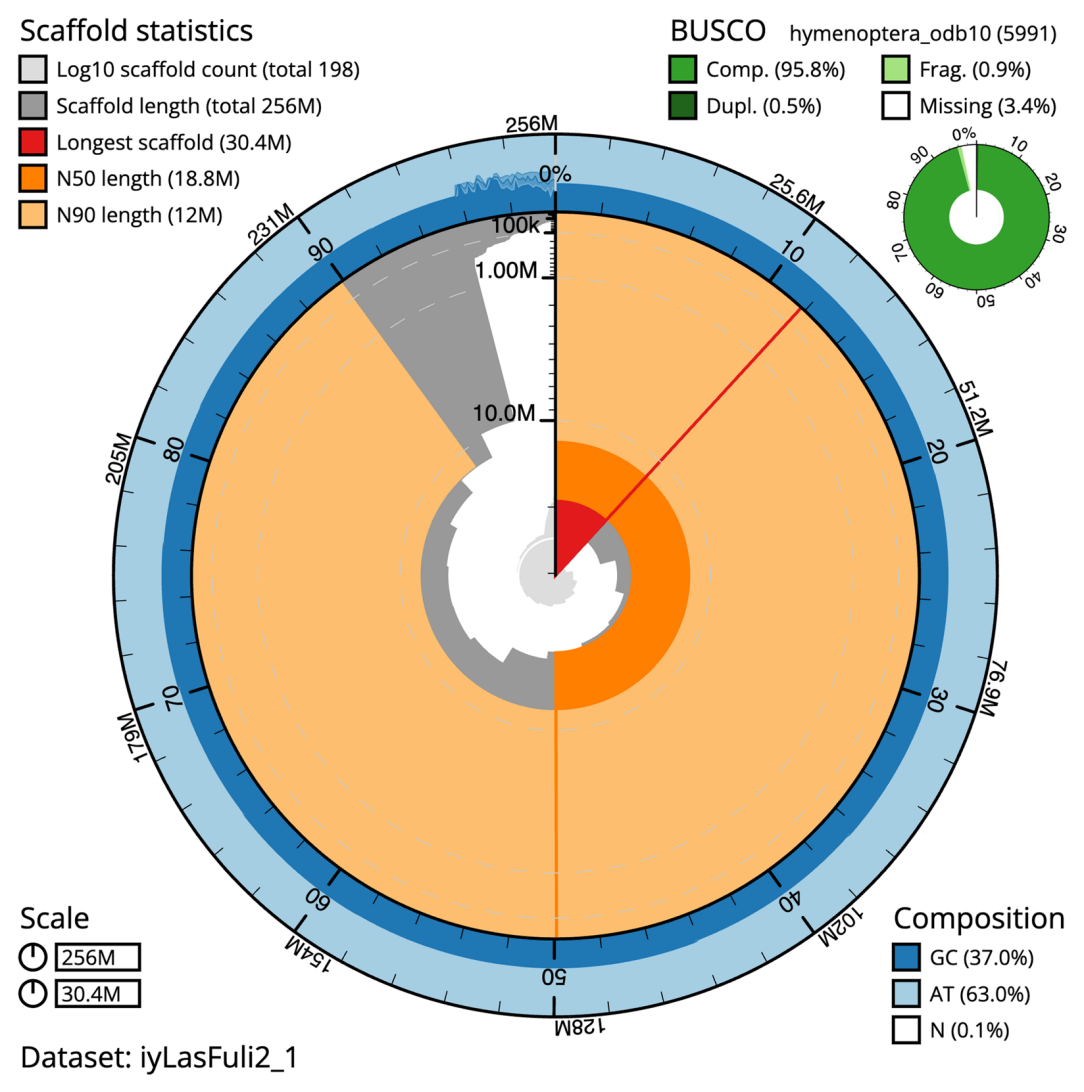
Genome assembly of *Lasius fuliginosus*, iyLasFuli2.1: metrics. The BlobToolKit snail plot provides an overview of assembly metrics and BUSCO gene completeness. The circumference represents the length of the whole genome sequence, and the main plot is divided into 1,000 equal-sized bins around the circumference. The outermost blue tracks display the distribution of GC, AT, and N percentages across the bins. Scaffolds are arranged clockwise from longest to shortest and are depicted in dark grey. The longest scaffold is indicated by the red arc, and the deeper orange and pale orange arcs represent the N50 and N90 lengths. A light grey spiral at the centre shows the cumulative scaffold count on a logarithmic scale. A summary of complete, fragmented, duplicated and missing BUSCO genes in the hymenoptera_odb10 set is shown in the top right. An interactive version of this figure is available at https://blobtoolkit.genomehubs.org/view/iyLasFuli2_1/dataset/iyLasFuli2_1/snail.

**Figure 3.**
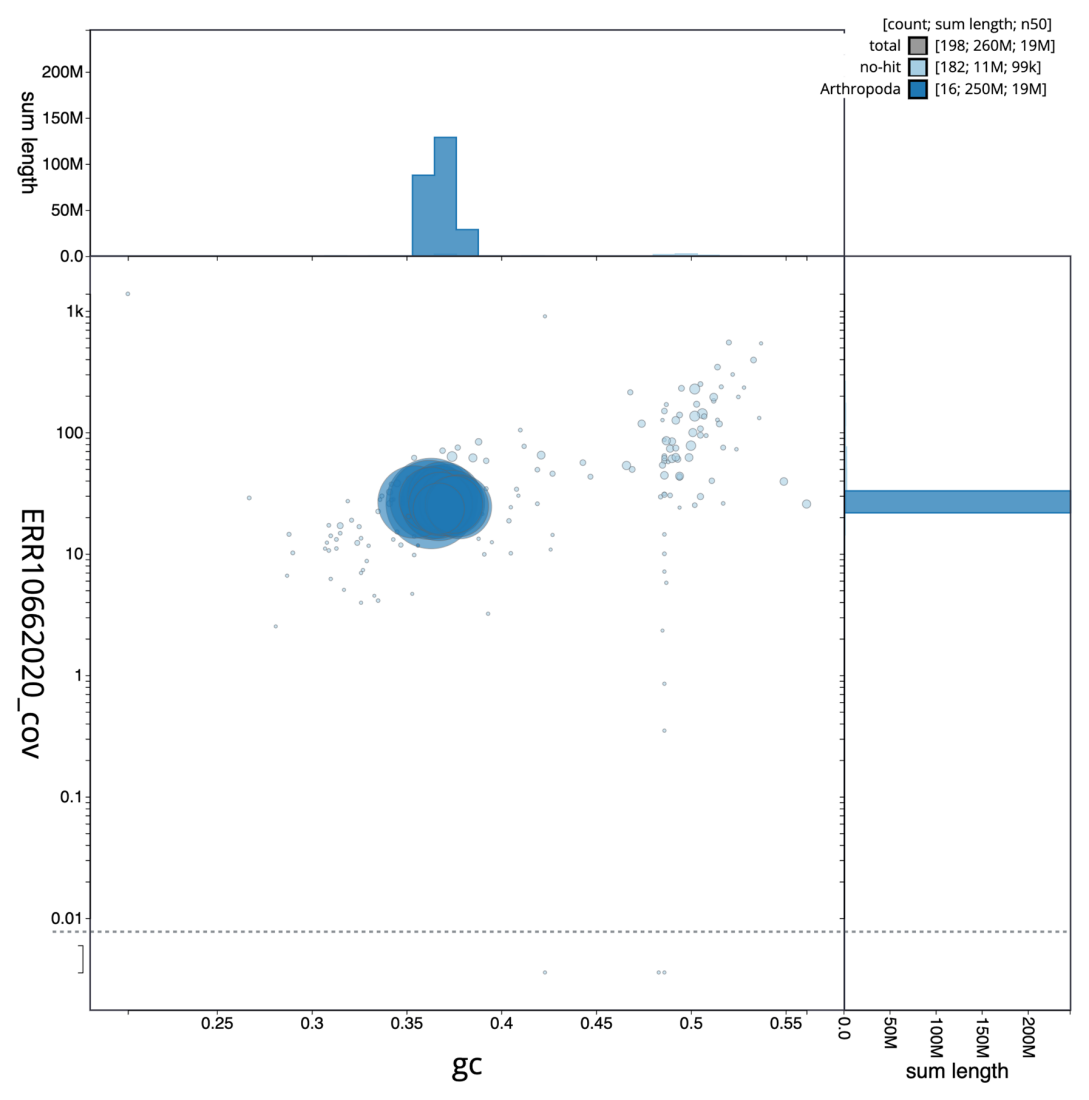
Genome assembly of *Lasius fuliginosus*, iyLasFuli2.1: BlobToolKit GC-coverage plot. Sequences are coloured by phylum. Circles are sized in proportion to sequence length. Histograms show the distribution of sequence length sum along each axis. An interactive version of this figure is available at https://blobtoolkit.genomehubs.org/view/iyLasFuli2_1/dataset/iyLasFuli2_1/blob.

**Figure 4.**
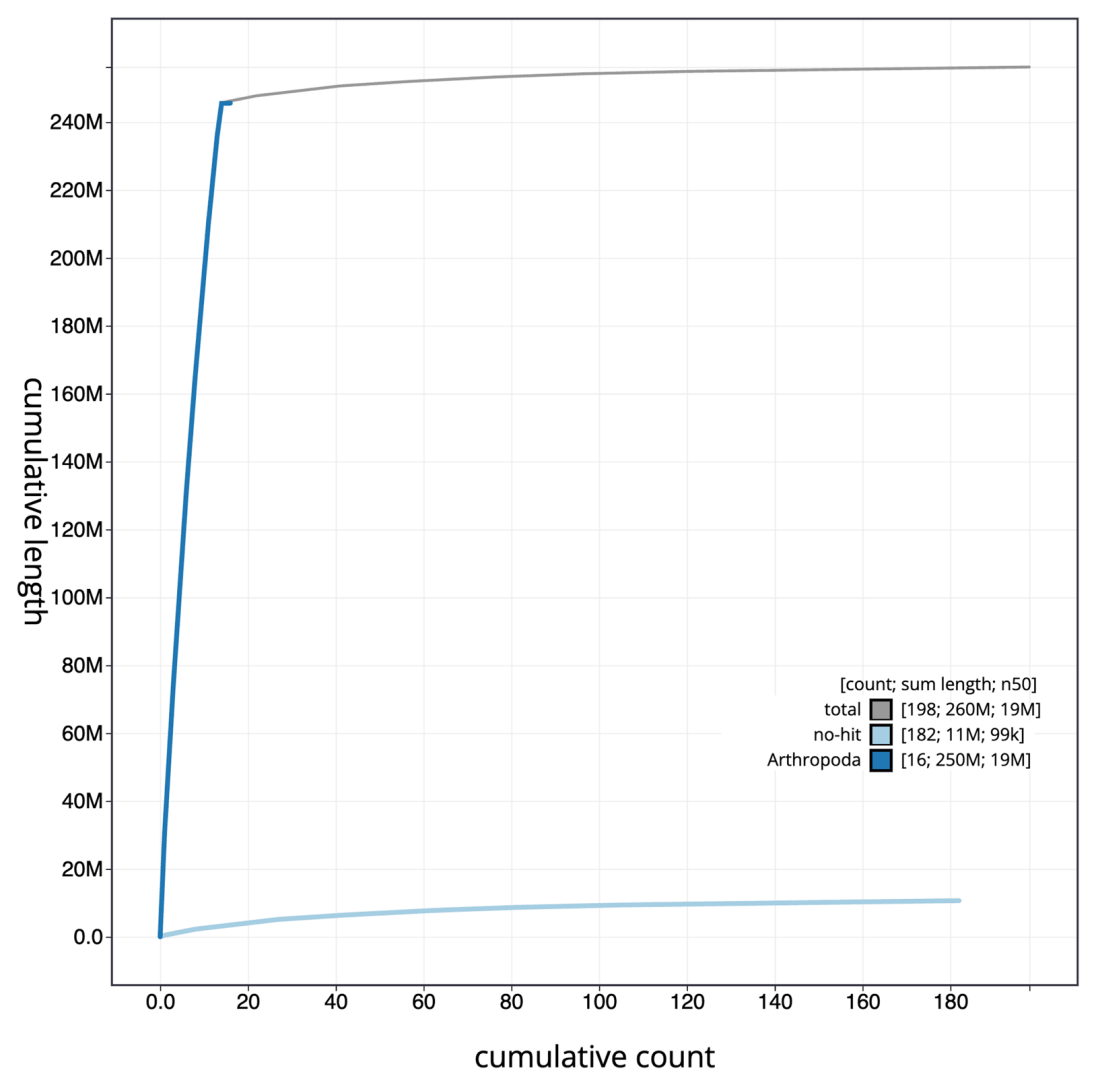
Genome assembly of *Lasius fuliginosus*, iyLasFuli2.1: BlobToolKit cumulative sequence plot. The grey line shows cumulative length for all sequences. Coloured lines show cumulative lengths of sequences assigned to each phylum using the buscogenes taxrule. An interactive version of this figure is available at https://blobtoolkit.genomehubs.org/view/iyLasFuli2_1/dataset/iyLasFuli2_1/cumulative.

**Figure 5.**
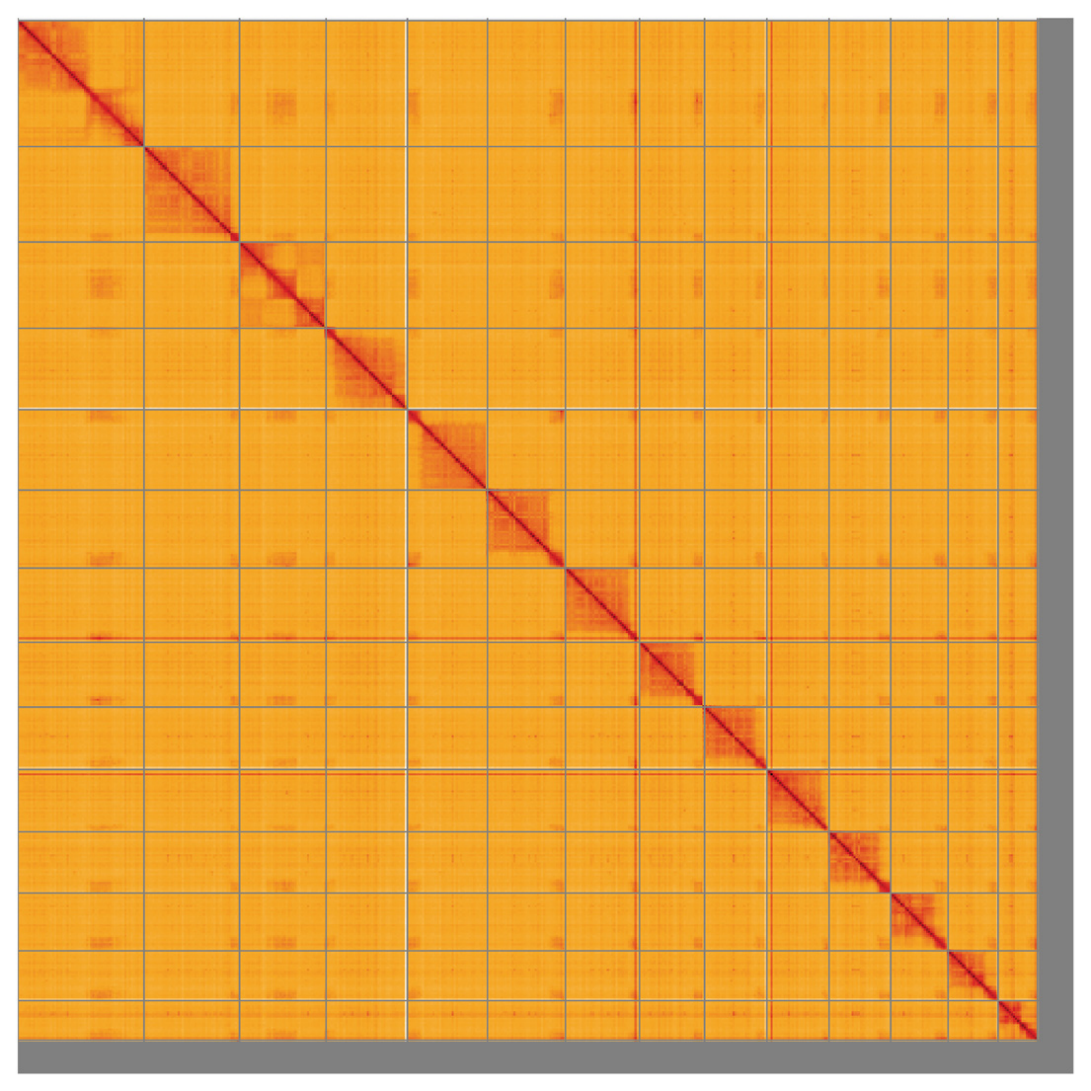
Genome assembly of *Lasius fuliginosus*, iyLasFuli2.1: Hi-C contact map of the iyLasFuli2.1 assembly, visualised using HiGlass. Chromosomes are shown in order of size from left to right and top to bottom. An interactive version of this figure may be viewed at https://genome-note-higlass.tol.sanger.ac.uk/l/?d=A4t4mW78SDa-FXRqR6TdAw.

The final assembly has a total length of 256.2 Mb in 197 sequence scaffolds with a scaffold N50 of 18.8 Mb ( Table 1). The snail plot in Figure 2 provides a summary of the assembly statistics, while the distribution of assembly scaffolds on GC proportion and coverage is shown in Figure 3. The cumulative assembly plot in Figure 4 shows curves for subsets of scaffolds assigned to different phyla. Most (95.81%) of the assembly sequence was assigned to 14 chromosomal-level scaffolds. Chromosome-scale scaffolds confirmed by the Hi-C data are named in order of size ( Figure 5; Table 2). The following regions are of uncertain order and orientation: Chromosome 14, in the region 1.6–4.5 Mb. While not fully phased, the assembly deposited is of one haplotype. Contigs corresponding to the second haplotype have also been deposited. The mitochondrial genome was also assembled and can be found as a contig within the multifasta file of the genome submission.

**Table 1.** Genome data for *Lasius fuliginosus*, iyLasFuli2.1.

Project accession data		
Assembly identifier	iyLasFuli2.1	
Species	*Lasius fuliginosus*	
Specimen	iyLasFuli2	
NCBI taxonomy ID	231986	
BioProject	PRJEB57896	
BioSample ID	Genome sequencing: SAMEA10200642 Hi-C scaffolding: SAMEA10200641	
Isolate information	iyLasFuli2: whole organism (genome sequence) iyLasFuli1: whole organism (Hi-C sequencing)	
Raw data accessions		
PacificBiosciences Sequel IIe	ERR10662020	
Hi-C Illumina	ERR10614878	
Genome assembly		
Assembly accession	GCA_949152495.1	
*Accession of alternate* *haplotype*	GCA_949152525.1	
Span (Mb)	256.2	
Number of contigs	971	
Number of scaffolds	197	
Longest scaffold (Mb)	30.36	
Assembly metrics *		*Benchmark*
Contig N50 length (Mb)	0.5	*≥ 1 Mb*
Scaffold N50 length (Mb)	18.8	*= chromosome N50*
Consensus quality (QV)	52.8	*≥ 40*
*k*-mer completeness	99.98%	*≥ 95%*
BUSCO **	C:95.8%[S:95.3%,D:0.5%], F:0.9%,M:3.3%,n:5,991	*S > 90%, D < 5%*
Percentage of assembly mapped to chromosomes	95.81%	*≥ 90%*
Sex chromosomes	None	*localised* *homologous pairs*
Organelles	Mitochondrial genome: 18.75 kb	*complete single* *alleles*

* Assembly metric benchmarks are adapted from Rhie *et al.* (2021) and the Earth BioGenome Project Report on Assembly Standards September 2024. ** BUSCO scores based on the hymenoptera_odb10 BUSCO set using version 5.3.2. C = complete [S = single copy, D = duplicated], F = fragmented, M = missing, n = number of orthologues in comparison. A full set of BUSCO scores is available at https://blobtoolkit.genomehubs.org/view/iyLasFuli2_1/dataset/iyLasFuli2_1/busco.

**Table 2.** Chromosomal pseudomolecules in the genome assembly of *Lasius fuliginosus*, iyLasFuli2.

INSDC accession	Name	Length (Mb)	GC%
OX424443.1	1	30.36	36.5
OX424444.1	2	23.02	36.0
OX424445.1	3	20.8	36.5
OX424446.1	4	19.55	36.5
OX424447.1	5	19.29	35.5
OX424448.1	6	18.82	37.0
OX424449.1	7	17.86	37.0
OX424450.1	8	15.62	37.5
OX424451.1	9	15.06	36.5
OX424452.1	10	15.0	38.0
OX424453.1	11	14.78	37.0
OX424454.1	12	13.86	37.5
OX424455.1	13	12.02	37.5
OX424456.1	14	9.47	36.5
OX424457.1	MT	0.02	20.5

The estimated Quality Value (QV) of the final assembly is 52.8 with *k*-mer completeness of 99.98%, and the assembly has a BUSCO v5.3.2 completeness of 95.8% (single = 95.3%, duplicated = 0.5%), using the hymenoptera_odb10 reference set ( *n* = 5,991).

Metadata for specimens, barcode results, spectra estimates, sequencing runs, contaminants and pre-curation assembly statistics are given at https://links.tol.sanger.ac.uk/species/231986.

## Methods

### Sample acquisition

Adult specimens of *Lasius fuliginosus* were collected from Dry Sandford Pit, Oxfordshire, UK (latitude 51.69, longitude –1.32) on 2021-03-30 by potting. The specimens were collected and identified by Liam Crowley (University of Oxford) and preserved on dry ice. One specimen (specimen ID Ox001082, ToLID iyLasFuli2) was used for PacBio HiFi sequencing, and another (specimen ID Ox001081, ToLID iyLasFuli1) was used for Hi-C sequencing.

### Nucleic acid extraction

The workflow for high molecular weight (HMW) DNA extraction at the Wellcome Sanger Institute (WSI) Tree of Life Core Laboratory includes a sequence of procedures: sample preparation and homogenisation, DNA extraction, fragmentation and purification. Detailed protocols are available on protocols.io ( Denton *et al.*, 2023b).

In sample preparation, the iyLasFuli2 sample was weighed and dissected on dry ice ( Jay *et al.*, 2023). Tissue from the whole organism was homogenised using a PowerMasher II tissue disruptor ( Denton *et al.*, 2023a).

HMW DNA was extracted in the WSI Scientific Operations core using the Automated MagAttract v2 protocol ( Oatley *et al.*, 2023). The DNA was sheared into an average fragment size of 12–20 kb in a Megaruptor 3 system ( Bates *et al.*, 2023). Sheared DNA was purified by solid-phase reversible immobilisation, using AMPure PB beads to eliminate shorter fragments and concentrate the DNA ( Strickland *et al.*, 2023). The concentration of the sheared and purified DNA was assessed using a Nanodrop spectrophotometer and Qubit Fluorometer and Qubit dsDNA High Sensitivity Assay kit. Fragment size distribution was evaluated by running the sample on the FemtoPulse system.

### Sequencing

Pacific Biosciences HiFi circular consensus DNA sequencing libraries were constructed according to the manufacturers’ instructions. DNA sequencing was performed by the Scientific Operations core at the WSI on a Pacific Biosciences Sequel IIe instrument. Hi-C data were also generated from remaining whole organism tissue of iyLasFuli1 using the Arima v2 kit. The Hi-C sequencing was performed using paired-end sequencing with a read length of 150 bp on the Illumina NovaSeq 6000 instrument.

### Genome assembly and curation

Assembly was carried out with Hifiasm ( Cheng *et al.*, 2021) and haplotypic duplication was identified and removed with purge_dups ( Guan *et al.*, 2020). The assembly was then scaffolded with Hi-C data ( Rao *et al.*, 2014) using YaHS ( Zhou *et al.*, 2023). The assembly was checked for contamination and corrected as described previously ( Howe *et al.*, 2021). The mitochondrial genome was assembled using MitoHiFi ( Uliano-Silva *et al.*, 2023), which runs MitoFinder ( Allio *et al.*, 2020) and uses these annotations to select the final mitochondrial contig and to ensure the general quality of the sequence.

Manual curation was performed using HiGlass ( Kerpedjiev *et al.*, 2018) and PretextView ( Harry, 2022).

### Evaluation of final assembly

A Hi-C map for the final assembly was produced using bwa-mem2 ( Vasimuddin *et al.*, 2019) in the Cooler file format ( Abdennur & Mirny, 2020). To assess the assembly metrics, the *k*-mer completeness and QV consensus quality values were calculated in Merqury ( Rhie *et al.*, 2020). This work was done using Nextflow ( Di Tommaso *et al.*, 2017) DSL2 pipelines “sanger-tol/readmapping” ( Surana *et al.*, 2023a) and “sanger-tol/genomenote” ( Surana *et al.*, 2023b). The genome was analysed within the BlobToolKit environment ( Challis *et al.*, 2020) and BUSCO scores ( Manni *et al.*, 2021) were calculated.

Table 3 contains a list of relevant software tool versions and sources.

**Table 3.** Software tools: versions and sources.

Software tool	Version	Source
BEDTools	2.30.0	https://github.com/arq5x/bedtools2
Blast	2.14.0	ftp://ftp.ncbi.nlm.nih.gov/blast/executables/blast+/
BlobToolKit	4.3.7	https://github.com/blobtoolkit/blobtoolkit
BUSCO	5.4.3 and 5.5.0	https://gitlab.com/ezlab/busco
bwa-mem2	2.2.1	https://github.com/bwa-mem2/bwa-mem2
Cooler	0.8.11	https://github.com/open2c/cooler
DIAMOND	2.1.8	https://github.com/bbuchfink/diamond
fasta_windows	0.2.4	https://github.com/tolkit/fasta_windows
FastK	427104ea91c78c3b8b8b49f1a7d6bbeaa869ba1c	https://github.com/thegenemyers/FASTK
GoaT CLI	0.2.5	https://github.com/genomehubs/goat-cli
Hifiasm	0.16.1-r375	https://github.com/chhylp123/hifiasm
HiGlass	1.11.6	https://github.com/higlass/higlass
HiGlass	44086069ee7d4d3f6f3f0012569789ec138f42b84 aa44357826c0b6753eb28de	https://github.com/higlass/higlass
MerquryFK	d00d98157618f4e8d1a9190026b19b471055b22e	https://github.com/thegenemyers/MERQURY.FK
MitoHiFi	2	https://github.com/marcelauliano/MitoHiFi
MultiQC	1.14, 1.17, and 1.18	https://github.com/MultiQC/MultiQC
NCBI Datasets	15.12.0	https://github.com/ncbi/datasets
Nextflow	23.04.0-5857	https://github.com/nextflow-io/nextflow
PretextView	0.2	https://github.com/sanger-tol/PretextView
purge_dups	1.2.3	https://github.com/dfguan/purge_dups
samtools	1.16.1, 1.17, and 1.18	https://github.com/samtools/samtools
sanger-tol/ genomeassembly	0.10.0	https://github.com/sanger-tol/genomeassembly
sanger-tol/ genomenote	1.1.1	https://github.com/sanger-tol/genomenote
sanger-tol/ readmapping	1.2.1	https://github.com/sanger-tol/readmapping
Seqtk	1.3	https://github.com/lh3/seqtk
Singularity	3.9.0	https://github.com/sylabs/singularity
TreeVal	1.0.0	https://github.com/sanger-tol/treeval
YaHS	yahs-1.1.91eebc2	https://github.com/c-zhou/yahs

### Wellcome Sanger Institute – Legal and Governance

The materials that have contributed to this genome note have been supplied by a Darwin Tree of Life Partner. The submission of materials by a Darwin Tree of Life Partner is subject to the **‘Darwin Tree of Life Project Sampling Code of Practice’**, which can be found in full on the Darwin Tree of Life website here. By agreeing with and signing up to the Sampling Code of Practice, the Darwin Tree of Life Partner agrees they will meet the legal and ethical requirements and standards set out within this document in respect of all samples acquired for, and supplied to, the Darwin Tree of Life Project.

Further, the Wellcome Sanger Institute employs a process whereby due diligence is carried out proportionate to the nature of the materials themselves, and the circumstances under which they have been/are to be collected and provided for use. The purpose of this is to address and mitigate any potential legal and/or ethical implications of receipt and use of the materials as part of the research project, and to ensure that in doing so we align with best practice wherever possible. The overarching areas of consideration are:

•   Ethical review of provenance and sourcing of the material

•   Legality of collection, transfer and use (national and international)

Each transfer of samples is further undertaken according to a Research Collaboration Agreement or Material Transfer Agreement entered into by the Darwin Tree of Life Partner, Genome Research Limited (operating as the Wellcome Sanger Institute), and in some circumstances other Darwin Tree of Life collaborators.

## Data Availability

European Nucleotide Archive: *Lasius fuliginosus* (jet ant). Accession number PRJEB57896; https://identifiers.org/ena.embl/PRJEB57896. The genome sequence is released openly for reuse. The *Lasius fuliginosus* genome sequencing initiative is part of the Darwin Tree of Life (DToL) project. All raw sequence data and the assembly have been deposited in INSDC databases. The genome will be annotated using available RNA-Seq data and presented through the Ensembl pipeline at the European Bioinformatics Institute. Raw data and assembly accession identifiers are reported in Table 1.

## Author information

Members of the University of Oxford and Wytham Woods Genome Acquisition Lab are listed here: https://doi.org/10.5281/zenodo.7125292.

Members of the Darwin Tree of Life Barcoding collective are listed here: https://doi.org/10.5281/zenodo.4893703.

Members of the Wellcome Sanger Institute Tree of Life Management, Samples and Laboratory team are listed here: https://doi.org/10.5281/zenodo.10066175.

Members of Wellcome Sanger Institute Scientific Operations: Sequencing Operations are listed here: https://doi.org/10.5281/zenodo.10043364.

Members of the Wellcome Sanger Institute Tree of Life Core Informatics team are listed here: https://doi.org/10.5281/zenodo.10066637.

Members of the Tree of Life Core Informatics collective are listed here: https://doi.org/10.5281/zenodo.5013541.

Members of the Darwin Tree of Life Consortium are listed here: https://doi.org/10.5281/zenodo.4783558.
