# Supplementary material for: The genome sequence of the jet ant, Lasius fuliginosus (Latreille, 1798)
Source: Wellcome Open Res. 2024 Nov 8;9:668. [Version 1] doi: 10.12688/wellcomeopenres.23347.1 (PMC11809159; doi:10.12688/wellcomeopenres.23347.1)
Supplement: Peer review history

# Reviewer report: Reviewer response for version 1

**DOI:** 10.21956/wellcomeopenres.25732.r112177

**Published:** 2024-12-19

**Authors:** Sara E Miller

**Affiliations:** 1 Department of Biology, University of Missouri St. Louis, Cornell University, St Louis, Missouri, USA

In this note, the authors present a new high quality nuclear and mitochondrial genome assembly for the jet ant *Lasius fuliginosus *(Hymenoptera: Formicidae). This species is notable for its interesting life history in which queens form new colonies through temporary social parasitism. This genome assembly will add to the growing number of ant genomes, providing new insight into the evolution of sociality.

 The genome was constructed for single female using Pacific Biosciences HiFi circular consensus DNA libraries, sequenced on the Biosciences SEQUEL IIe, and scaffolded with Hi-C data. Raw data and assembly methods are available. The final assembly is 256.2 Mb in length with a scaffold N50 of 18.8 Mb. The genome assembly has 95.8% coverage of BUSCO genes. The genome assembly does not include an annotation.

 While overall a nice report of a high-quality ant genome, I request that the authors to please cite the original primary literature, rather than AntWiki in the background information on the organism. This supports original researchers of this species.

Are sufficient details of methods and materials provided to allow replication by others?

Yes

Is the rationale for creating the dataset(s) clearly described?

Yes

Are the datasets clearly presented in a useable and accessible format?

Yes

Are the protocols appropriate and is the work technically sound?

Yes

Reviewer Expertise:

Hymenoptera genetics and genomics, evolutionary ecology

I confirm that I have read this submission and believe that I have an appropriate level of expertise to confirm that it is of an acceptable scientific standard, however I have significant reservations, as outlined above.

# Reviewer report: Reviewer response for version 1

**DOI:** 10.21956/wellcomeopenres.25732.r114097

**Published:** 2024-12-18

**Authors:** Lamjed Mansour

**Affiliations:** 1 King Saud University, Riyadh, Riyadh Province, Saudi Arabia

The paper represents a successful sequencing and assembling of *Lasius fuliginosus* genome, as a contribution to the Darwin Tree of Life Project.

 This ant is known for its temporary social parasitism and complex nest-building behavior in deciduous woodlands which is widespread across Europe and parts of Asia. The assembled genome, derived from a female specimen collected in Oxfordshire, UK, spans 256.2 megabases and includes 14 chromosomal pseudomolecules. The mitochondrial genome of 18.75 kb was also sequenced. Author used a combination of Pacific Biosciences HiFi long reads and Hi-C scaffolding data, and achieved a high-quality assembly (QV of 52.8 and 95.8% BUSCO ). This genome of *L. fuliginosus* is publicly available through specified databases, provides a valuable resource for future studies in ant genetics, evolution, and social behavior. The assembly meets current genomic standards.

 The methodology is sound and adheres to standard protocols for genome sequencing and assembly.

**I have minor corrections:**

- Please used appropriate scientific papers as references in addition to Antwiki database which was used a lot as reference.
- Please clarify if the 95.8% BUSCO completeness is considered typical/acceptable for Hymenoptera.
- Please Include comparison to any existing genomic resources for related Lasius species
- Please add a brief discussion of potential applications of this genome sequence
- Include acknowledgment of any limitations in the assembly or analysis

Are sufficient details of methods and materials provided to allow replication by others?

Yes

Is the rationale for creating the dataset(s) clearly described?

Yes

Are the datasets clearly presented in a useable and accessible format?

Yes

Are the protocols appropriate and is the work technically sound?

Yes

Reviewer Expertise:

Immunogenetics and molecular evolution

I confirm that I have read this submission and believe that I have an appropriate level of expertise to confirm that it is of an acceptable scientific standard.

# Reviewer report: Reviewer response for version 1

**DOI:** 10.21956/wellcomeopenres.25732.r113101

**Published:** 2024-12-13

**Authors:** Joshua D Gibson

**Affiliations:** 1 Georgia Southern University, Statesboro, USA

The data note “The genome sequence of the jet ant, *Lasius fuliginosus* (Latreille, 1798)” by Liam Crowley provides the sequence and assembly of the genome for a previously unsequenced ant species as part of the Darwin Tree of Life Project. The sequence is based on a single female worker ant and the assembly was scaffolded using Hi-C chromosomal conformation data from a second female worker ant. The assembly includes 14 chromosome length scaffolds as well as the assembled mitochondrial genome. The assembly appears to be relatively complete based on *k*-mer and BUSCO completeness tests. All data is freely available and Table 1 provides a very easy overview of the appropriate accessions. Similarly, all software sources and versions are clearly provided as well.

 There are only a few relatively small suggestions I have to improve the data note.

 In the Background section I would recommend removing the broad citations to AntWiki, 2024 where possible and instead substitute the appropriate literature citations. Many of these are available in the AntWiki article, but proper attribution to the sources is best practice.

 The samples and their processing in the methods could be clarified. There are two samples, ToLID iyLasFuli1 and iyLasFuli2. iyLasFuli1 was used for Hi-C sequencing and iyLasFuli2 was used for HiFi sequencing. However, in the Nucleic acid extraction section, only iyLasFuli2 is noted and it is stated that the specimen was dissected. Then under the Sequencing section the “remaining whole organism tissue” of iyLasFuli1 is noted as the source of the Hi-C data. This brings up a few questions/comments that would best be clarified in the methods.

- It seems that listing both specimens and their extraction methods in the Nucleic acid extraction section would make the most sense rather than including iyLasFuli1 in the Sequencing section.
- What was dissected in the iyLasFuli2 sample and how does this relate to the subsequent sentence stating that tissue from the whole organism was homogenized? The Jay *et al* 2023 citation doesn’t include any more specifics of *what* may be dissected, just protocols for *if *dissection was performed.
- Similarly, was iyLasFuli1 dissected in some way, and if so, how? If not, what does the “remaining whole organism” refer to?

These are relatively minor, but clarifying these methods will ensure that the underlying data is fully utilizable for future analyses.

Are sufficient details of methods and materials provided to allow replication by others?

Yes

Is the rationale for creating the dataset(s) clearly described?

Yes

Are the datasets clearly presented in a useable and accessible format?

Yes

Are the protocols appropriate and is the work technically sound?

Yes

Reviewer Expertise:

Evolutionary genetics, insects, physiology, behavior

I confirm that I have read this submission and believe that I have an appropriate level of expertise to confirm that it is of an acceptable scientific standard.
